# Competent Non‐Native Alternative Host Reduces Infection Success in a Generalist Parasite

**DOI:** 10.1002/ece3.73645

**Published:** 2026-05-27

**Authors:** Markéta Ondračková, Lukáš Vetešník, Veronika Bartáková, Michal Janáč

**Affiliations:** ^1^ Institute of Vertebrate Biology of the Czech Academy of Sciences Brno Czech Republic

**Keywords:** alternative host, dilution effect, eye fluke, non‐native species, parasite success

## Abstract

Successful parasite proliferation depends on eco‐evolutionarily stable host–parasite relationships, which may be disrupted by the introduction of non‐native alternative hosts, particularly in the case of the generalist parasites adapted to multiple host species. Here, we experimentally analysed how the addition of a non‐native alternative host affects the infection success of a larval generalist trematode, *Diplostomum pseudospathaceum*. We assessed parasite abundance, the proportion of successfully established metacercariae, and host‐dependent parasite growth in the principal ( *Abramis brama* ), the native alternative ( *Rutilus rutilus* ), and the non‐native alternative ( *Lepomis gibbosus* ) fish intermediate hosts. Across all experiments, *A. brama* showed the highest susceptibility to *D. pseudospathaceum*, with significantly lower abundances in both alternative hosts. This supports the view that evolutionary compatibility and host–parasite coadaptation in generalists play a more decisive role in shaping infection patterns than ecological factors such as host abundance or encounter rates. Multi‐host experiments revealed a significant reduction in the proportion of successfully established metacercariae, particularly in combinations of native hosts with non‐native *L. gibbosus* , indicating a dilution effect. Moreover, *L. gibbosus* exhibited a pronounced response to infection, developing stronger cataract than native hosts. Cataract formation was also more likely at lower infection intensities, indicating a lower threshold for pathology in the non‐native host without a shared evolutionary history with the parasite, compared to long‐term coexisting hosts. Metacercariae differed subtly yet significantly in size and organ dimensions among host species, demonstrating that host identity can affect larval morphology. Overall, our results demonstrate that the presence of alternative hosts, especially the non‐native ones, can reduce parasite transmission through encounter dilution and suboptimal infection, even when those hosts are susceptible, confirming that dilution effects are not restricted to non‐competent or resistant hosts.

## Graphical abstract

In this study, we experimentally assessed how the addition of a non‐native alternative host affects the infection success of the larval generalist trematode, *Diplostomum pseudospathaceum*, in fish intermediate hosts. Our results demonstrate that the presence of alternative hosts, especially the non‐native ones, can reduce parasite transmission through encounter dilution and suboptimal infection, even when those hosts are susceptible, confirming that dilution effects are not restricted to non‐competent or resistant hosts.

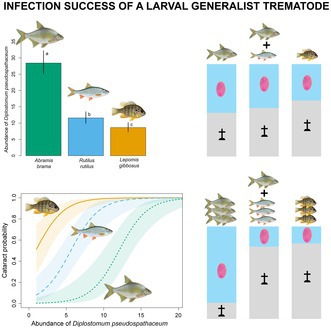

## 1 Introduction

Understanding the mechanisms governing host–parasite interactions is essential for addressing ecological challenges such as the spread of infectious diseases, biological control, species invasions, and climate‐driven biotic shifts (Hoberg and Brooks 2015). Introduced species can affect all levels of ecological organization by imposing novel species interactions and altering existing ones (Sax et al. 2005). Among these effects, the disruption of native host–parasite relationships by non‐native species represents a subtle but increasingly recognized phenomenon (Goedknegt et al. 2016; Poulin 2017; Chalkowski et al. 2018).

Parasite proliferation depends on eco‐evolutionary stable host–parasite relationships that continuously ensure growth, survival, reproduction and dispersal (Wells and Flynn 2022). Non‐native species may alter the abundance of native hosts and prevalence of pathogens through various direct and indirect mechanisms affecting disease transmission (Burghardt et al. 2010; Chinchio et al. 2020; Atkinson and Savage 2023). For example, competition or predation may reduce native host densities below thresholds for effective pathogen transmission (Slade et al. 2023), while resistant or less‐competent hosts can generate encounter‐dilution effects, reducing rates of parasitic infections manifested in native host species (Johnson et al. 2019, Venesky et al. 2014, Keesing and Ostfeld 2021). Conversely, competent non‐native hosts may amplify parasite prevalence and abundance, potentially leading to increased infection rates ‘spillback’ (Kelly et al. 2009).

Host specificity is a key determinant of parasite distribution, local abundance, and evolutionary dynamics (Combes 2001; Krasnov et al. 2005; Mouillot et al. 2006). Specialist parasites (specializing in one or a few host species) prioritize host quality (Manzoli et al. 2021) but are less resilient during environmental disruptions affecting the host community structure (Auld et al. 2017). On the other hand, generalist parasites exhibit adaptability to multiple host species, often in unequal proportions and with context‐dependent variability (Carvalho et al. 2024). Generalists benefit from broad host availability, which, however, may result in suboptimal exploitation of the host (Lievens et al. 2018). Host specificity plays a central role in determining the strength and occurrence of the dilution effect in native hosts. High host specificity often limits dilution because transmission stages are rarely wasted on unsuitable hosts (Ostfeld and Keesing 2012; Zhou et al. 2024), while the dilution effect is most pronounced in generalist parasites when resistant or non‐competent hosts are introduced (Kopp and Jokela 2007; Thieltges et al. 2009; Ahn and Goater 2021).

Within generalist parasite populations, principal hosts support the majority of individuals, while alternative hosts range from highly competent species that facilitate persistence to poorly competent species acting as epidemiological dead‐ends (Poulin and Mouillot 2004; Doherty and Poulin 2022). The introduction of a non‐native species increases the availability of alternative hosts, but their utilization by generalist parasites may be influenced by multiple factors, including parasite density in the principal host(s) and the surrounding environment (Combes 2001; Searle et al. 2016), ecological or phylogenetic relatedness of alternative hosts (Lootvoet et al. 2013), host community composition (Holt et al. 2003), and environmental conditions (Silva et al. 2025). While infection of principal hosts typically enhances parasite survival and fitness (Manzoli et al. 2018), a broad host range enables generalist parasites to persist in diverse environments and adapt to fluctuating ecological conditions (Lootvoet et al. 2013; Manzoli et al. 2021).

Host density further influences parasite transmission dynamics. Epidemiological theory predicts that the infrapopulation abundance should increase asymptotically with host density due to higher contact rates between hosts and transmission stages (Buck and Lutterschmidt 2017). However, this applies primarily to directly transmitted parasites. For parasites that have to actively seek for their hosts, such as mobile free‐living stages of parasites with complex life cycles, additional hosts may dilute infection risk by distributing transmission stages among a greater number of potential hosts (Buck et al. 2017). Nevertheless, empirical evidence for encounter‐dilution effects in parasites with complex life cycles remains limited (Buck and Lutterschmidt 2017; Gendron and Marcogliese 2017).

Eye flukes, the larvae (metacercariae) of the *Diplostomum* trematodes (Digenea: Diplostomidae), are important pathogens infecting fish eyes. Molecular approaches have delineated over 40 species/species‐level lineages (Schwelm et al. 2021). Several species are known to affect fish behaviour (Crowden and Broom 1980; Seppälä et al. 2004, 2005; Klemme and Karvonen 2016), reproduction success (Nezhybová et al. 2020), and mortality (Pennycuick 1971; Michálková and Ondračková 2014). *Diplostomum pseudospathaceum* Niewiadomska, 1984 is a common species in Eurasia (Kudlai et al. 2017; Lebedeva et al. 2020), reproducing in fish‐eating birds (Laridae), with lymnaeid snails and various fish species serving as first and second intermediate hosts, respectively (Niewiadomska 2002). In fish, metacercariae infecting eye lenses often cause cataract at high intensities of infection, impairing vision (Karvonen and Marcogliese 2020).

Natural infections of *D. pseudospathaceum* have been confirmed in diverse fish orders (Behrmann‐Godel 2013; Georgieva et al. 2013; Kudlai et al. 2017; Locke et al. 2020; Unger et al. 2022; Kvach et al. 2023; Ondračková, Kvach, et al. 2025a), with considerably variable host susceptibility. Salmonids (Klemme et al. 2020; Karvonen et al. 2023; Mikheev and Pasternak 2023) and sticklebacks (Kalbe and Kurtz 2006; Scharsack and Kalbe 2014), show relatively high vulnerability, while for example roach *Rutilus rutilus* (Linnaeus, 1758) or bleak *Alburnus alburnus* (Linnaeus, 1758) (Cyprinidae) appear less vulnerable (Kudlai et al. 2017). Similarly, invasive fish species in Europe also differ in susceptibility to *D. pseudospathaceum*, ranging from high (*Ameiurus* spp.; Ondračková, Kvach, et al. 2025a), through medium (*Neogobius* spp.; Kudlai et al. 2024), to low ( *Lepomis gibbosus* (Linnaeus, 1758); Kvach et al. 2023, Ondračková et al. 2023).

This study aims to investigate the infection success of *D. pseudospathaceum* in native and invasive fish species. Specifically, we tested (1) how invasive hosts influence the success of a generalist parasite when susceptible but suboptimal to the infection, particularly under varying host densities and (2) how the invasive hosts respond to infection by a parasite with which they share no common evolutionary history, compared to native alternative hosts. We first identified the composition of *Diplostomum* spp. in common fish species from small lentic water bodies in the Czech Republic to estimate the primary and secondary hosts of *D. pseudospathaceum* in the study area. Based on these results, we experimentally compared the parasite infection success (measured as intensity of infection and the proportion of successfully developed parasites), parasite morphological traits, and host response (cataract formation) in principal, native alternative and invasive alternative hosts. Combining field and experimental approaches, we evaluated whether invasive species act as competent hosts that contribute to parasite transmission or as evolutionary traps reducing parasite fitness.

## 2 Material and Methods

### 2.1 Collection and Identification of Parasites in Naturally Infected Fish

To identify the species distribution of *Diplostomum* spp. among the fish hosts in natural lentic habitats of the southeastern Czech Republic, and to estimate the host susceptibility to *D. pseudospathaceum*, we collected common fish species in lentic water bodies along the lower stretch of the Morava River (Czech Republic, Danube basin). Fish sampling was completed within a complex study focused on natural parasite species distribution among the native and non‐native fish species in the study area (Appendix S1). Immediately after collection, live fish were transported to the laboratory where they were examined for a presence of the eye‐fluke infection. Parasites were removed from the fish tissues (lens, vitreous humor) and preserved in 96% ethanol for species determination using molecular methods.

Following Faltýnková et al. (2022), sequencing of the ribosomal internal transcribed spacer cluster ITS1‐5.8S‐ITS2 and partial mitochondrial gene cytochrome *c* oxidase subunit I (COI) were used as the most commonly employed markers, with a number of corresponding sequences available in the GenBank database for a wide range of *Diplostomum* spp. To determine the species, a total of 129 individual parasites were used for molecular analyses. DNA was extracted using the innuPREP Forensic Kit (Analytik Jena, Germany). The internal transcribed spacer 1 (ITS1)‐specific primers BD1 (forward: 5′‐GTCGTAACAAGGTTTCCGTA‐3′) and 4S (reverse: 5′‐TCTAGATGCGTTCGAARTGTCGATG‐3′) (Bowles et al. 1993; Luton et al. 1992) were used for sequencing the ITS1‐5.8S‐ITS2 region under the conditions described in Dudliv et al. (2024). The diplostomid‐specific primers Plat‐diploCOX1F (5′‐CGT TTR AAT TAT ACG GAT CC‐3′) and Plat‐diploCOX1R (5′‐AGC ATA GTA ATM GCA GCA GC‐3′) were used to amplify COI (Moszczynska et al. 2009).

The PCR reaction mix had a total volume of 10 μL, comprising 4 μL of extracted DNA, 0.3 μL primer, 2 μL buffer A, 0.2 μL dNTPs, 0.2 μL MgCl_2_, 0.1 μL Taq polymerase and 2.9 μL ddH_2_O. The KAPA2G Robust Hot‐Star PCR Kit (Kapabiosystems, USA) was used for PCR analysis. Amplification was performed in a Mastercycler ep gradient S thermocycler (Eppendorf, Germany) with an annealing temperature of 57°C and 50°C, respectively. The PCR product was then purified using an ExoSAP‐IT kit (Affymetrix Inc., Santa Clara, USA), according to the manufacturer's protocol, and sequenced commercially at Eurofins Genomics Germany GmbH (Germany). The sequences were edited and aligned using Geneious v. 9.0.5 software (Kearse et al. 2012). Ambiguous results were found for *Diplostomum* from the vitreous humour of *Perca fluviatilis* Linnaeus, 1758 and *Gymnocephalus cernuus* (Linnaeus, 1758), showing affiliation to *Diplostomum* sp. lineage 3 sensu Faltýnková et al. (2022) according to ITS1‐5.8S‐ITS2 rRNA and *Diplostomum* sp. lineage 4 sensu Faltýnková et al. (2022) according to COI. As the main aim of this study was not focused on the taxonomy of *Diplostomum* species and because percid hosts were not included in the experimental trials, no further detailed analysis aiming to resolve this taxonomic bias was performed and the species is here denoted as *Diplostomum* sp. lineage 3/4 sensu Faltýnková et al. (2022). Based on the results, three species vulnerable to the eye fluke (*Diplostomum* spp.) infection were selected for consequent experimental testing: *Abramis brama* Linnaeus, 1758 representing a native preferred principal host, *Rutilus rutilus* representing a native alternative host and *Lepomis gibbosus* representing a non‐native alternative host.

### 2.2 Animal Collection and Breeding

Adult specimens of *A. brama* (total length TL: 255–295 mm), *R. rutilus* (TL: 128–158 mm) (Cyprinidae) and *L. gibbosus* (TL: 108–118 mm) (Centrarchidae) were collected by electrofishing in natural water bodies in the Morava River basin (Czech Republic) and transported alive to the institutional facility. Both *A. brama* and *R. rutilus* are native to Europe. Both species are known to host metacercariae of the eye‐fluke parasite *Diplostomum pseudospathaceum* (Kudlai et al. 2017; Locke et al. 2015). *Lepomis gibbosus* is non‐native in Europe, having been introduced from North America in the late nineteenth century. The species is susceptible to *Diplostomu*m infection in its native range (Désilets et al. 2013; Chapman et al. 2015; Locke et al. 2010), as well as to the infection of *D. pseudospathaceum* in Europe, though with a much lower load (Kvach et al. 2023).

For fish breeding, semi‐natural spawning was used for obtaining juveniles of *L. gibbosus* . Males of *L. gibbosus* build a nest on the bottom substrate and guard the egg clutch for 5–6 days before the fingerlings hatch, and for a further 10 days after hatching. Males spawn repeatedly in the same nest with one or more females (Kottelat and Freyhof 2007). Therefore, a single adult male and two adult females were placed into outdoor fibreglass tubs equipped with gravel as a spawning substrate and artificial plants as a refuge and left to naturally spawn. Three replicates were performed. Three weeks after fingerling release, adult fish were removed from the tubs, and juvenile fish were fed by plankton naturally established in the water and supplemented with freshly hatched nauplii of *Artemia* sp. After 25 days, fish were fed two times per day with frozen adults of *Artemia* sp. and the commercially dry pellet.

To obtain juvenile *A. brama* and *R. rutilus* , fish were separated according to sex into two well‐aerated tanks and were stimulated for ovulation/spermiation by carp pituitary and by subsequently increasing the water temperature to 20°C. Oocytes of ovulating females and sperm were sampled according to Gela et al. (2009). Juvenile fish were placed in aquaria with oxygenated water and were fed three times per day with freshly hatched nauplii of *Artemia* sp. (24 h from salt‐water incubation; Sanders, Ogden, USA) from hatching until 30 days. After 30 days, fish were fed two times per day with frozen adults of *Artemia* sp., the commercially dry pellet and flakes. Fish were kept under controlled conditions until experimental infection.

For fish experimental infection, freshwater snails ( *Lymnaea stagnalis* Linnaeus, 1758) were collected in Vlkovský Pond (Lužnice basin, Czech Republic) and checked for natural infection by *D. pseudospathaceum*. The snails were individually placed into the 250 mL containers with tap water and those releasing cercariae of *Diplostomum*‐type were selected for further procedures. Cercariae released from 10 snails were fixed in 96% ethanol and parasite species identity was confirmed by genetic analysis using COI and ITS1‐5.8S‐ITS2 following Georgieva et al. (2013). Occurrence of *D. pseudospathaceum* was confirmed for all ten samples.

### 2.3 Experimental Infection

**FIGURE 1:**
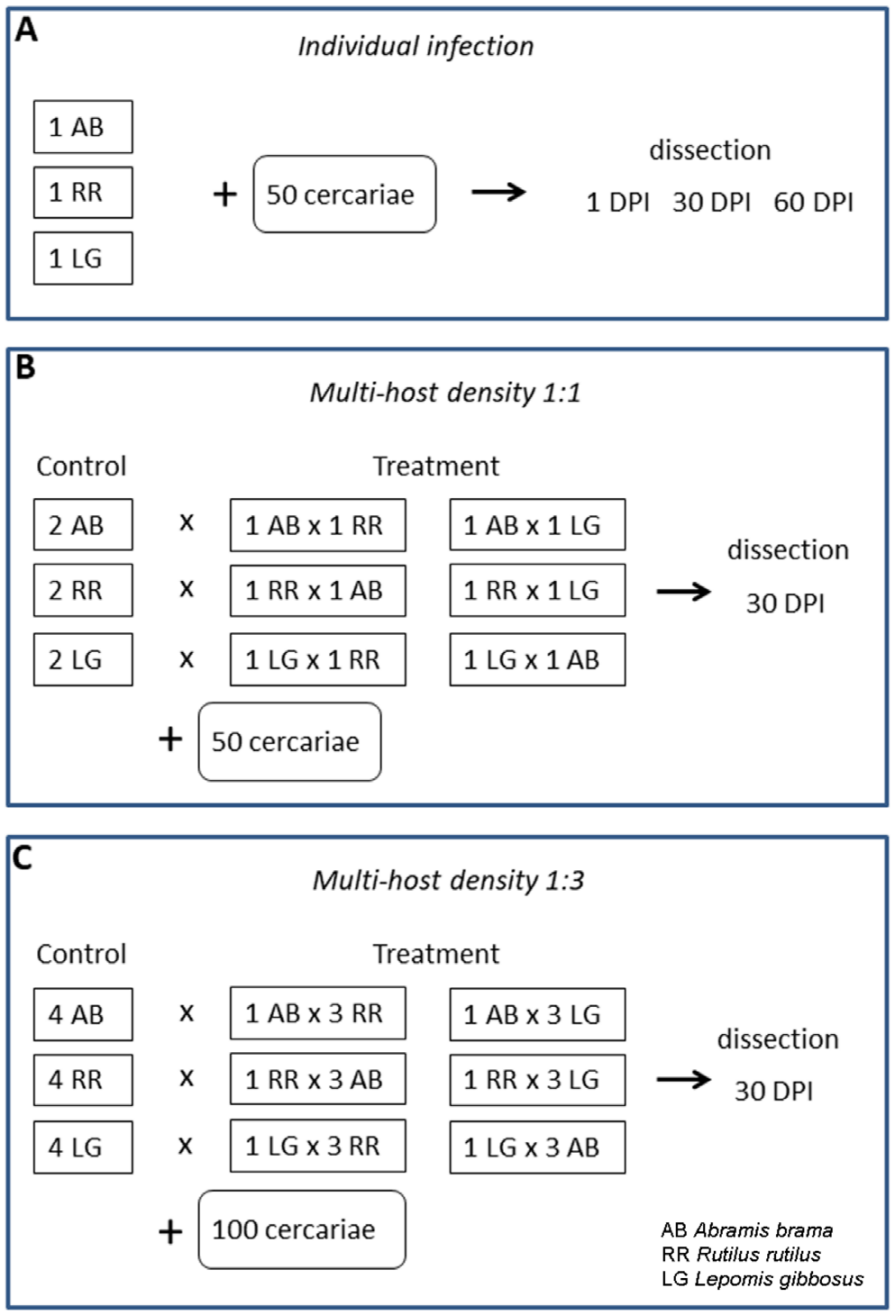
Scheme of experimental setups. AB— *Abramis brama* , RR— *Rutilus rutilus* , LG— *Lepomis gibbosus* .

Three experimental setups were conducted. The first setup, *Individual infection*, was performed for all three fish species to confirm fish species vulnerability to parasite infection, their response to the infection by cataract formation and preliminary determination of principal/alternative host species (Figure 1A). In multi‐host species systems, two experiments were conducted for each pair of fish species in order to test whether alternative host reduces infection success in principal (or more vulnerable alternative) host species, using setups with equal fish density, that is, *Multi‐host density 1:1* (Figure 1B), and with dominance of either principal or alternative fish host species, that is, *Multi‐host density 1:3* (Figure 1C).

Prior to the infection, ten infected snails were placed into one‐litre container with standing tap water for two hours to produce cercariae at room temperature. Cercarial density in the suspension that was used for fish infection was estimated from ten 0.1 mL sub‐samples from which the number of cercariae was counted under a binocular microscope (see Michálková and Ondračková 2014). Preliminary infection tests showed that the infection dose of 25 cercariae per fish was high enough to cause infection in all three fish species tested, yet the dose of 50 cercariae was low enough to minimize fish host mortality (note that juvenile fish were used for parasite infection). The fish were exposed to cercariae for one hour, with the water temperature in individual containers (250 mL)/setups corresponding to the temperature in aquaria where the fish were maintained before and after experimental infection, that is, 23°C for *Individual infection* (16th August) and *Multi‐host density 1:1* (18th August), and 20°C for *Multi‐host density 1:3* (6th September). After the parasite exposure, fish were removed from plastic containers and returned back into the aquaria. Fish of the same species and the same experimental setup were grouped together and kept in aquaria till dissection. Prior to dissection, all fish were anaesthetized by overdosing with clove oil in accordance with Law 246/1992 of the Czech Republic and standard length (SL, to the nearest mm) was measured.

For *Individual infection*, a single fish was exposed to 50 cercariae in 200 mL of standing tap water (*n* = 24/fish species). Eight fish per species were dissected 1 day post infection (DPI), 30 DPI and 60 DPI. Number of metacercariae in each lens was counted and cataract formation scored from 0–5: 0 = lens fully transparent, 1 = small localized opacity, usually in the close proximity of parasite/s, 2 = Opacity covers 26%–50% of the lens, 3 = Large opacity, covering 51%–75% of the lens, 4 = almost entire lens opaque, 5 = lens fully opaque (modified following Karvonen et al. 2004). Parasites obtained from fish dissected 60 DPI, expected to be fully developed, were preserved in hot formaldehyde, stained with iron acetocarmine (Georgiev et al. 1986), dehydrated in ethanol of increasing concentrations, and mounted in Canada balsam for permanent mounting on slides for further morphological examination. In multi‐host systems, experimental infection was conducted for each pair of fish species, i. e. *A. brama* × *R. rutilus* (AB × RR), *A. brama* × *L. gibbosus* (AB × LG), and *R. rutilus* × *L. gibbosus* (RR × LG). As a control, conspecific pairs of each fish species underwent the same infection procedure. In the experiment with equal host density ‐ *Multi‐host density 1:1*, two fish, either multi‐species treatment or single species control, were placed into the plastic container with 50 cercariae and 200 mL of water in 12 replicates for all combinations. In the experiment with high host density ‐ *Multi‐host density 1:3*, four fish individuals, including one of one species and three of another species in multi‐species treatments, and four individuals in a single species control, were exposed to 100 cercariae in 400 mL of water, ensuring the same parasite density as in *Multi‐host density 1:1*. Six replicates were performed for heterospecific and three for conspecific treatments. For both setups, fish were dissected 30 DPI and number of live metacercariae counted in each lens. While no mortality occurred in *Individual infection* setup, two *A. brama* died in the treatment LG × AB prior the termination of the experiment in *Multi‐host density 1:1* setup. In *Multi‐host density 1:3* setup, one *R. rutilus* died in the control (4 RR), two *R. rutilus* died in the treatment 1AB × 3RR, four *A. brama* died in the control (4 AB) and two *A. brama* died in the treatment 1RR × 3AB.

### 2.4 Morphometric Analysis of Parasites

Morphometric measurements were completed for metacercariae isolated from five each of *A. brama* , *R. rutilus* and *L. gibbosus* obtained from *Individual infection* setup, 60 DPI, stained in iron acetocarmine and mounted in Canada balsam (see above). For measurements, an Olympus BX51 light microscope (Olympus Optical Co., Tokyo, Japan) equipped with phase‐contrast, differential interference contrast (DIC) optics, and a digital image analysis system OLYMPUS cellSens Standard digital image analysis package (Olympus Optical Co., Hamburg, Germany) was used. Eleven parameters were measured (in μm), including length and width of oral and ventral suckers, pharynx, and holdfast organ, length of pseudosuckers and body area. The body area was obtained in the image‐analysis software (OLYMPUS cellSens Standard) by tracing the full body contour, with the software subsequently computing the corresponding planar area. For statistical analysis, the mean length of both pseudosuckers was used.

### 2.5 Data Analysis

For all models that contained multiple predictors, best models were always selected using a backward stepwise approach with likelihood ratio tests (LR tests) used to compare two nested models (LR tests results confirmed by model selection using AICc values [not shown]; Zuur et al. 2009). Tukey HSD approach was conducted to control for type II error in multiple post hoc pairwise comparisons in all models (using functions glht and mcp from *multcomp* package, Hothorn et al. 2008).

Generalized linear model (GLM) was used to describe the effects of host species and days post infection (DPI) on the abundance of *D. pseudospathaceum*. Full model included host species, DPI and their interaction as predictors and the parasite abundance (i.e., the number of parasites per fish) as response variable (negative binomially distributed).

When analyzing the effects of host species and DPI on cataract stage score (0–5), fish dissected at DPI = 1 were removed from the analysis (too soon for cataract to develop). Generalized linear mixed model (GLMM, binomial distribution) was used on the remaining data set, with the full model containing host species, DPI and their interaction as predictors, fish individual as random effect and cataract stage as response variable (binomially distributed). Furthermore, the effect of parasite abundance on cataract formation probability was tested using GLM (Bernoulli distribution), with the full model containing host species, abundance of *D. pseudospathaceum* and their interaction as predictors.

For multi‐host systems, GLM was used to describe the effect of experimental setups (*Multi‐host density 1:1* and *1:3*) on parasite abundance (negative binomially distributed), with separate models conducted for each host species. Dunnett approach was conducted to control for type II error in post hoc pairwise comparisons between the control (i.e., single species) and the treatment (all other multi‐species combinations) using functions glht and mcp from *multicomp* package (Hothorn et al. 2008). In order to show whether the introduction of a non‐native species affects the proportion of successfully established parasites in multi‐host systems, we estimated the parasite success as a percentage of a number of live metacercariae extracted from fish lens out of the number of cercariae used for experimental infection in particular treatments. Differences in the proportion of successfully established parasites between fish species were tested by chi‐squared test.

To detect potential differences in morphology of metacercariae of *D. pseudospathaceum* among host species, the multiple analysis of variance (MANOVA) was used. For easier visualization of these inter‐host differences, we conducted principal component analysis (PCA) to find main gradients in measured morphological characteristics and reduce the number of dimensions. All morphological variables were standardized (mean = 0, SD = 1) prior to insertion in the PCA. Analysis of variance (AN) with Tukey HSD post hoc tests were used to find inter‐host differences in particular characteristics and in the axis coordinates. Association between intensity of parasite infection and values of parasite morphological parameters was tested using Spearman correlation rank test. All analyses were performed using the R software package v. 3.0.3 (R Core Team 2023).

## 3 Results

### 3.1 *Natural Distribution of* Diplostomum spp. *Among Fish Hosts*

**FIGURE 2:**
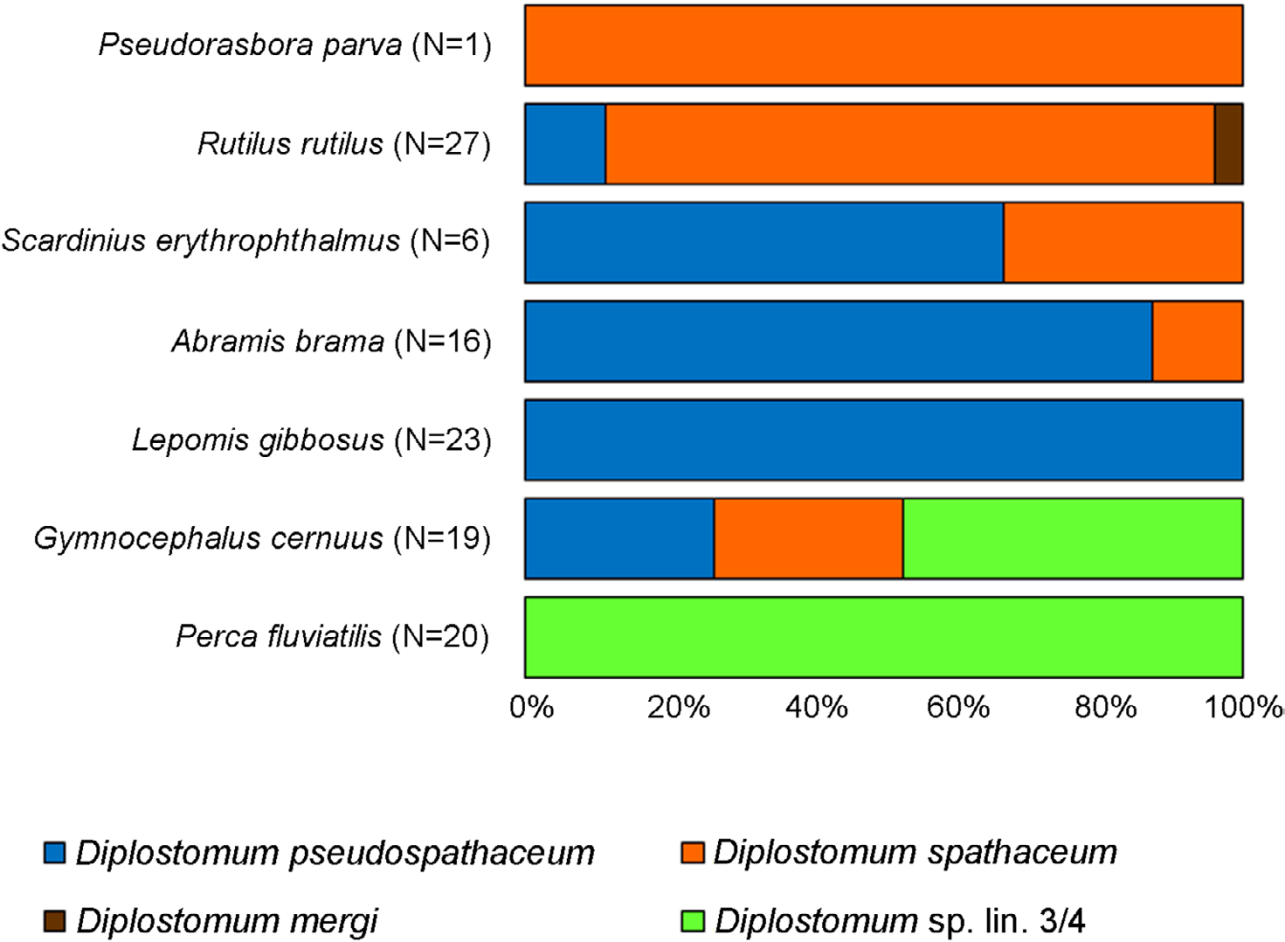
Proportion of different species of *Diplostomum* from seven infected fish species collected in lentic water bodies in the lower Morava River basin. The total number of successfully sequenced parasites per species is shown in parentheses. Parasite species identification was based on molecular analysis using ribosomal gene cluster ITS1‐5.8S‐ITS2 and mitochondrial cytochrome *c* oxidase subunit I.

Out of eleven fish species examined, seven were naturally infected with metacercariae of *Diplostomum* spp. (Appendix S1). Four species were distinguished in the sample of 111 parasites successfully sequenced (86% success). *Diplostomum pseudospathaceum* was found most frequently, being located in lenses of six fish host species: *R. rutilus* , *A. brama* , *Scardinius erythrophthalmus* (Linnaeus, 1758), *Pseudorasbora parva* Temnick and Schlegel, 1846, *L. gibbosus* , and *G. cernuus* . High susceptibility to *D. pseudospathaceum* was observed in *A. brama* , representing 88% of molecularly identified specimens. *Abramis brama* showed the highest prevalence and mean abundance (6.92) for the eye flukes of the genus *Diplostomum* in general. For the non‐native fish species *L. gibbosus* , *D. pseudospathaceum* was the only *Diplostomum* species detected in the study area, but its abundance was relatively low (0.28) compared with *A. brama* (see Appendix S1 for additional details). *Diplostomum spathaceum* (Rudolphi, 1819) was found in lenses of *R. rutilus* , *A. brama* , *S. erythrophthalmus* , and *G. cernuus* , with the highest infection parameters found in *R. rutilus* . *Diplostomum* sp. lineage 3/4 *sensu* Faltýnková et al. (2022) was found in the vitreous humor of *P. fluviatilis* and *G. cernuus* , and it was the only species found in *P. fluviatilis* . In addition, a single specimen of *Diplostomum mergi* Dubois, 1932 was detected in *R. rutilus* (Figure 2).

### 3.2 Host Vulnerability to Experimental Infection

**FIGURE 3:**
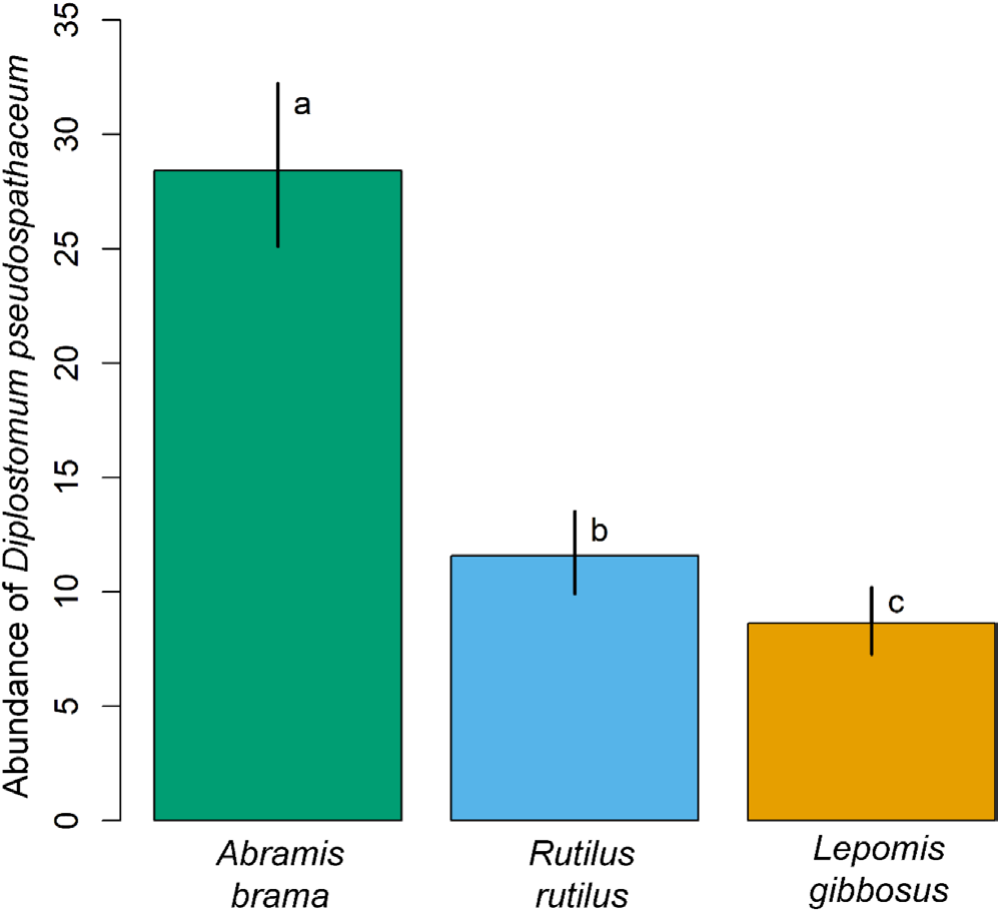
Mean (bars) and 95% CI estimates (vertical lines) of the infection of *Diplostomum pseudospathaceum* in three host species. Hosts sharing the same small case letter do not differ significantly from each other.

All fish individually exposed to cercariae were infected with at least two metaceraciae of *D. pseudospathaceum* (Table 1). Parasite abundance significantly differed among the hosts (GLM, d.f. = 269; LR test *p* < 0.001) and there was no significant effect of interaction (LR test, *p* = 0.923) or DPI (LR test, *p* = 0.144). Mean parasite abundance in *A. brama* was 28.4 ± 7.6 and more than doubled that observed in *R. rutilus* and *L. gibbosus* (Figure 3, GLM, post hoc comparisons, both *p* < 0.001), further confirming *A. brama* as the principle host species and the latter two species as the alternative hosts for *D. pseudospathaceum*. Parasite abundance in the non‐native alternative host, *L. gibbosus* , (8.6 ± 3.7) was significantly lower than that in the native alternative host, *R. rutilus* , (11.6 ± 5.3) (Table 1, Figure 3; GLM, post hoc comparison, *p* = 0.031).

**TABLE 1:** Number of fish (*N*), their standard length (SL, in mm) ± S.D. and abundance of *Diplostomum pseudospathaceum* (mean, range) in the three experimental setups.

	*N*	Fish SL	Parasite abundance
**Individual infection setup**			
*Abramis brama*	24	33 ± 4	28.4 (15–44)
*Rutilus rutilus*	24	39 ± 5	11.6 (2–26)
*Lepomis gibbosus*	24	35 ± 2	8.6 (3–18)
**Multi‐host density 1:1 setup**			
Control			
*Abramis brama* (AB × AB)	24	34 ± 2	13.2 (5–24)
*Rutilus rutilus* (RR × RR)	24	41 ± 2	8.5 (1–22)
*Lepomis gibbosus* (LG × LG)	24	32 ± 2	3.4 (0–9)
Treatment			
*Abramis brama* (AB × RR)	12	32 ± 2	15.6 (8–23)
*Abramis brama* (AB × LG)	12	34 ± 2	14.2 (10–19)
*Rutilus rutilus* (RR × AB)	12	42 ± 3	8.2 (2–16)
*Rutilus rutilus* (RR × LG)	12	43 ± 2	7.1 (1–16)
*Lepomis gibbosus* (LG × AB)	12	31 ± 2	6.2 (1–13)
*Lepomis gibbosus* (LG × RR)	12	33 ± 2	6.8 (3–11)
**Multi‐host density 1:3 setup**			
Control			
*Abramis brama* (4 AB)	8	32 ± 2	20.6 (9–25)
*Rutilus rutilus* (4 RR)	11	35 ± 2	4.8 (2–11)
*Lepomis gibbosus* (4 LG)	12	35 ± 1	1.9 (0–9)
Treatment			
*Abramis brama* (1 AB × 3 RR)	6	32 ± 2	11.0 (3–22)
~ *Rutilus rutilus*	16	35 ± 2	4.1 (1–11)
*Abramis brama* (1 AB × 3 LG)	6	33 ± 2	13.7 (4–23)
~ *Lepomis gibbosus*	18	35 ± 2	1.4 (0–4)
*Rutilus rutilus* (1 RR × 3 AB)	6	34 ± 2	2.7 (1–4)
~ *Abramis brama*	16	32 ± 2	10.6 (3–23)
*Rutilus rutilus* (1 RR × 3 LG)	6	34 ± 2	1.7 (0–6)
~ *Lepomis gibbosus*	18	34 ± 2	1.1 (0–4)
*Lepomis gibbosus* (1 LG × 3 AB)	6	31 ± 2	6.2 (1–13)
~ *Abramis brama*	18	32 ± 2	12.6 (4–22)
*Lepomis gibbosus* (1 LG × 3 RR)	6	33 ± 2	6.8 (3–11)
~ *Rutilus rutilus*	18	35 ± 2	4.6 (0–14)

**FIGURE 4:**
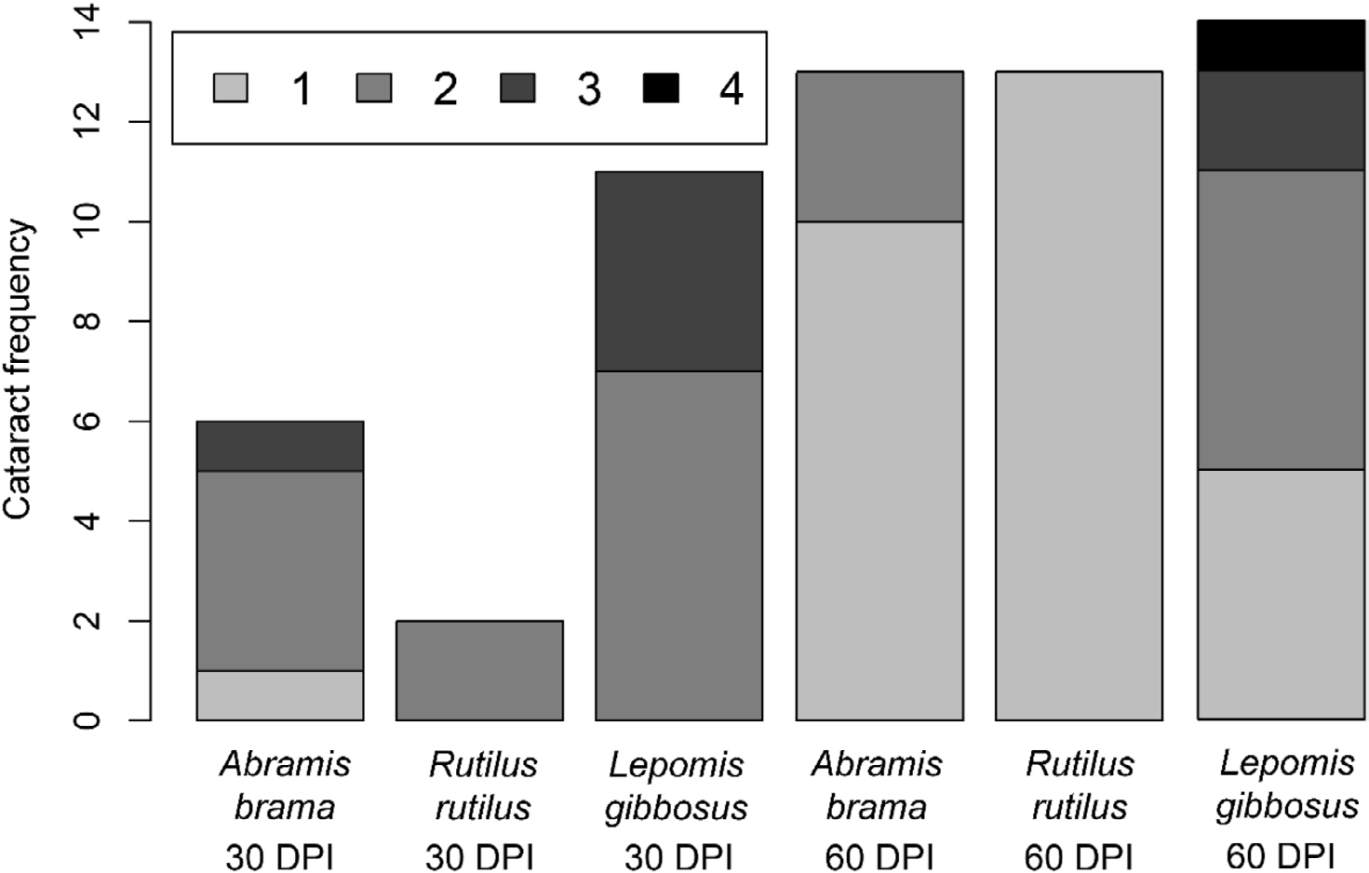
Overall incidence (total bar heights, out of 16 fish) and stage‐incidence (shade heights) of cataract based on the infection of *Diplostomum pseudospathaceum* in three fish host species 30 and 60 days post infection (DPI).

Cataract was observed in all fish species 30 and 60 DPI. The cataract score significantly differed among the hosts (GLMM, *n* = 96; LR test *p* < 0.001) and varied between 0–3 in *A. brama* , 0–2 in *R. rutilus* and 0–5 in *L. gibbosus* . There was no significant effect of interaction (LR test, *p* = 0.228) or DPI (LR test, *p* = 0.143, AIC 214.55 vs. 214.69). The cataract score was significantly higher in *L. gibbosus* than in *R. rutilus* and *A. brama* (GLMM post hoc comparisons, *p* < 0.001 and *p* = 0.029), with no significant difference between the two latter hosts (GLMM post hoc comparison, *p* = 0.277, Figure 4). A closer look at the data shows that cataract was more frequent in the non‐native *L. gibbosus* compared to the native hosts at 30 DPI, but this difference diminished 60 DPI. However, cataracts in *L. gibbosus* reached higher scores (prevailing score 2 and 3) at both 30 and 60 DPI. Among the native hosts, these scores (2 and 3) were observed almost exclusively at 30 DPI, though at comparatively lower frequency than in *L. gibbosus* , whereas at 60 DPI score 1 was consistently predominant (Figure 4).

**FIGURE 5:**
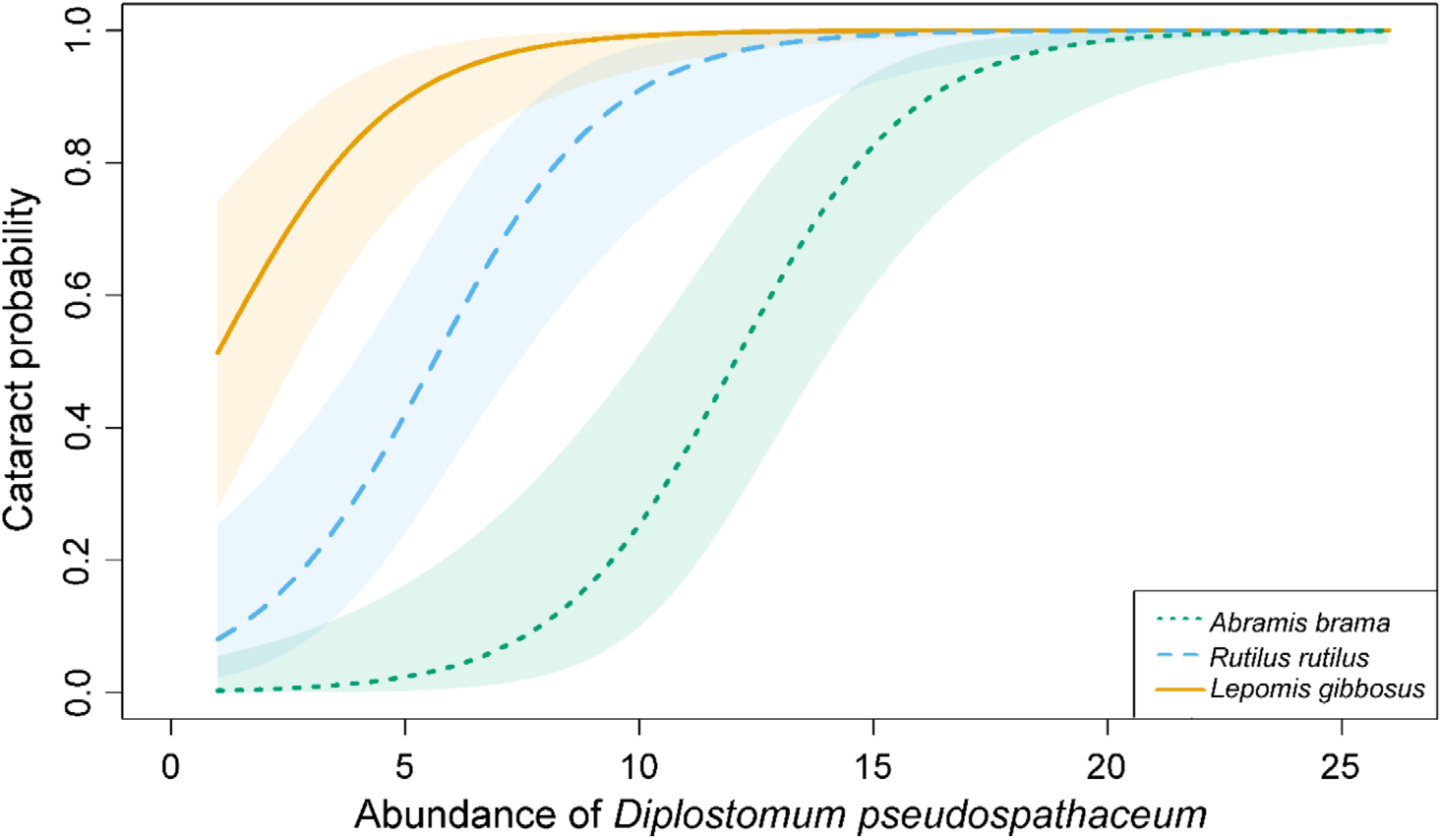
Probability of cataract formation with increasing abundance of *Diplostomum pseudospathaceum* in lens of experimentally infected *Abramis brama* (bluish green dotted line), *Rutilus rutilus* (sky blue dashed line), and *Lepomis gibbosus* (dark yellow solid line; generalized linear model, Bernoulli distribution). Shaded area corresponds to confidence intervals.

While the abundance of *D. pseudospathaceum* affected the probability of cataract formation in a similar way for all three host species (non‐significant effect of the interaction, GLM, LR test = 0.771), the probability clearly increased with the parasite abundance and differed among host species (GLM, LR tests both *p* < 0.001; Figure 5). The lowest infection intensities were sufficient to form a cataract in *L. gibbosus* , followed by *R. rutilus* and *A. brama* , with the differences being significant among all three hosts (GLM, post hoc comparisons, *p* = 0.001, *p* < 0.001 and *p* = 0.004; Figure 5).

### 3.3 Infection Success in Multi‐Host Systems—1:1

**FIGURE 6:**
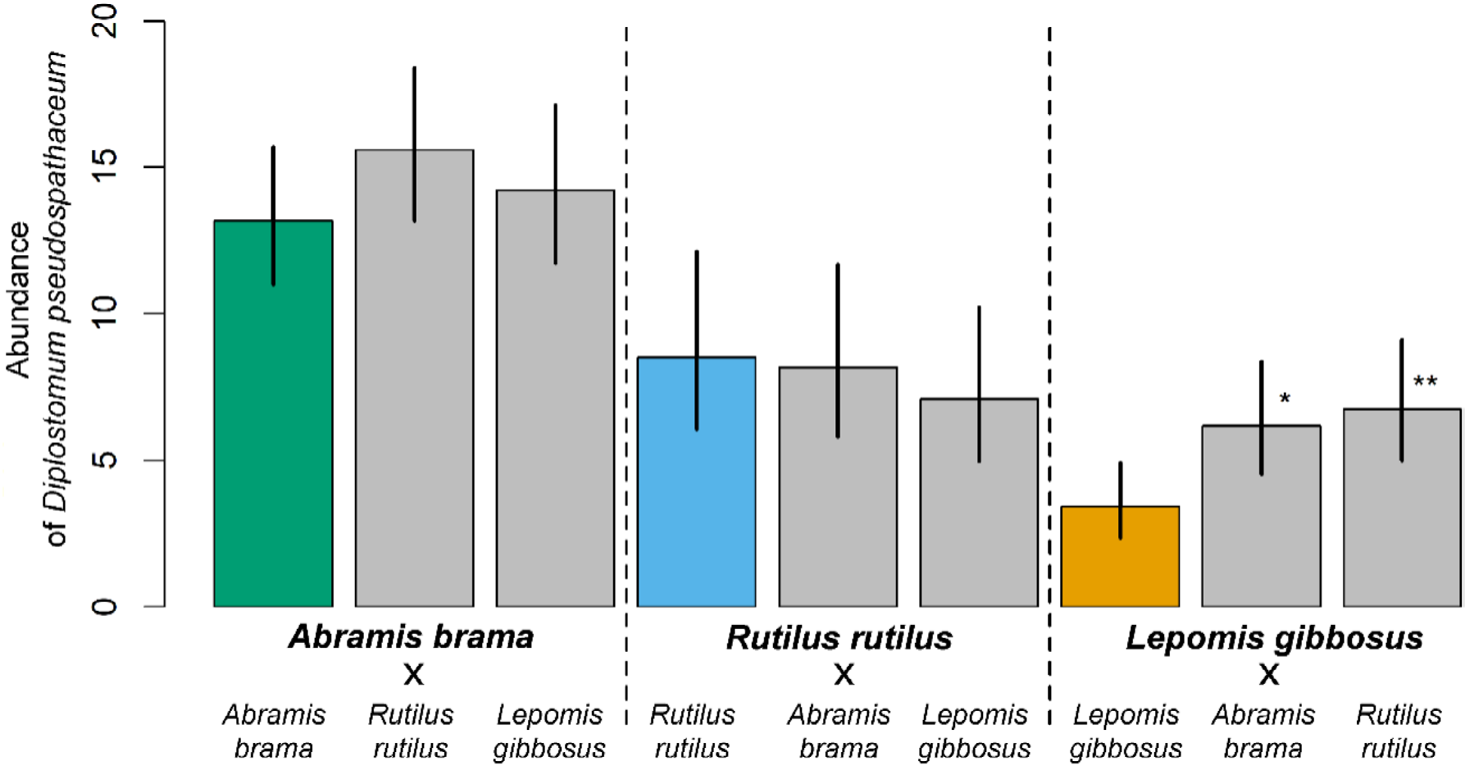
Abundance of *Diplostomum pseudospathaceum* in single host species (control; colour bars) and multi‐species (treatment; grey bars) in Multi‐host density 1:1 setup. Mean (bars) and 95% CI estimates (vertical lines) of the infection of *Diplostomum pseudospathaceum* calculated separately for three host species are presented. Significant differences from respective control monospecific setup are marked by symbols (* = 0.01 < *p* < 0.05; ** = 0.001 < *p* < 0.01).

Addition of a single host did not change the parasite abundance in either the primary or secondary native fish host when compared to conspecific infections (GLM; *A. brama* : d.f. = 2,31, *p* = 0.397; *R. rutilus* : d.f. = 2,33, *p* = 0.755; Figure 6). The joint exposure to cercariae, however, significantly increased the parasite abundance in the non‐native *L. gibbosus* compared to conspecific (control) groups (GLM, d.f. = 2,33, *p* = 0.011) in both LG × AB and LG × RR treatments (GLM post hoc comparisons, *p* = 0.029 and 0.009 for treatments with *A. brama* and *R. rutilus* , respectively; Figure 6).

In single species infections of this multi‐host system, 52.7%, 34.0% and 13.7% of cercariae successfully infected hosts and developed to metacercariae in AB × AB, RR × RR and LG × LG treatments, respectively. Addition of the native alternative host to the principal host (RR × AB) did not significantly affect the overall proportion of established parasites in the treatment (i.e., 52.7% vs. 47.5%; chi‐test, χ = 1.93, *p* = 0.164). On the other hand, addition of the non‐native alternative host decreased almost twice the proportion of established parasites in the treatment with *A. brama* (AB × LG) (i.e., 52.7% vs. 39.3%; χ = 13.60, *p* < 0.001), while the decrease was insignificant (though close to significance) in the treatment with *R. rutilus* (i.e., 34.0% vs. 27.7%; χ = 3.54, *p* = 0.060).

### 3.4 Infection Success in Multi‐Host Systems—1:3

**FIGURE 7:**
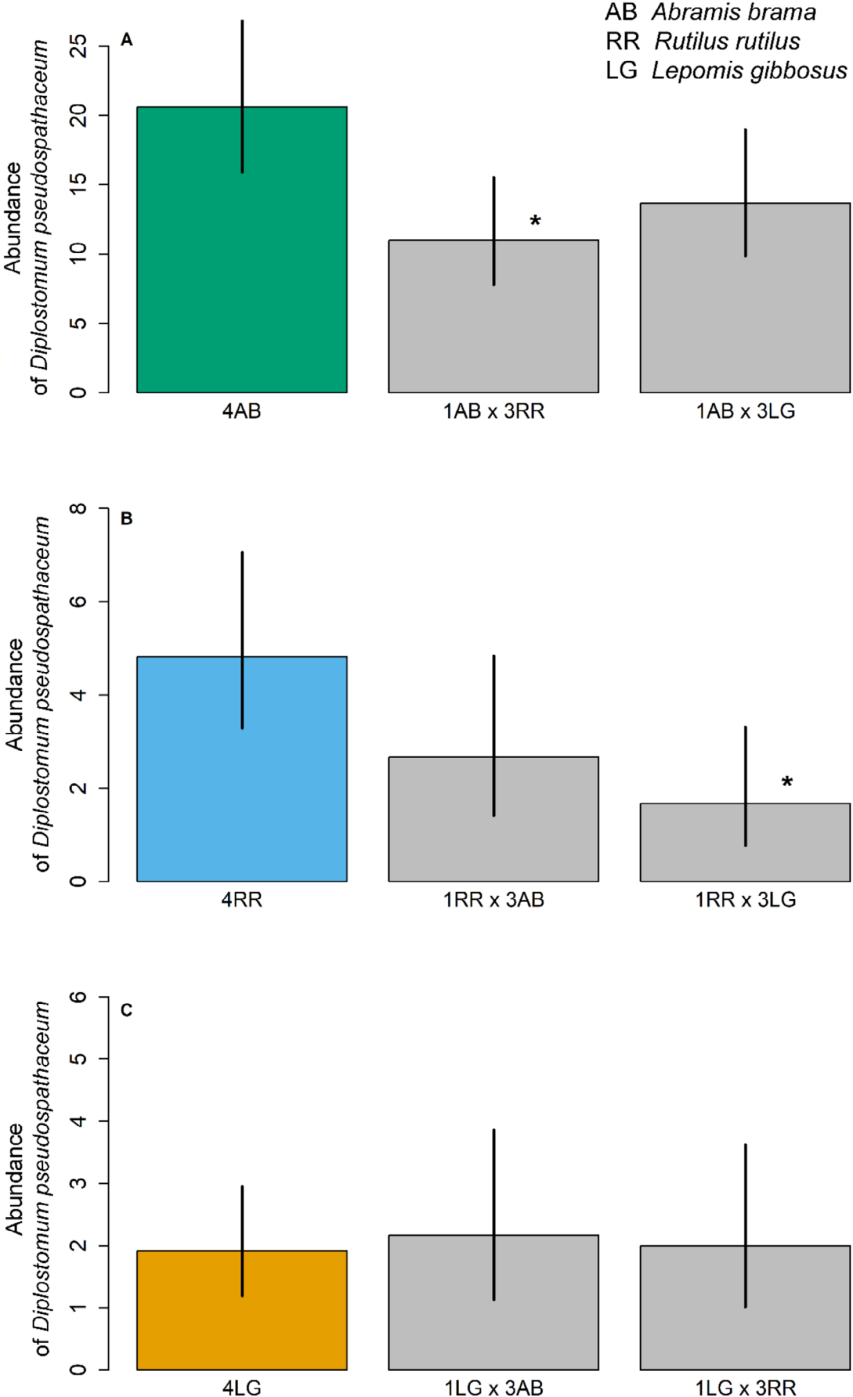
Abundance of *Diplostomum pseudospathaceum* in single host species (control; colour bar) and multi‐species (treatment; grey bars) in Multi‐host density 1:3 setup. Mean (bars) and 95% CI estimates (vertical lines) of the infection of *Diplostomum pseudospathaceum* were calculated separately for (A) *Abramis brama* (AB, green), (B) *Rutilus rutilus* (RR, blue), and (C) *Lepomis gibbosus* (LG, yellow). Significant differences from respective control monospecific setup are marked by symbols (* = 0.01 < *p* < 0.05).

In the high host density experimental setup, significant differences in parasite abundance were observed in both *A. brama* (GLM, d.f. = 2,17, *p* = 0.014) and *R. rutilus* (GLM, d.f. = 2,20, *p* = 0.006) but not in *L. gibbosus* (GLM, df = 2,21, *p* = 0.940; Figure 7). Decreased parasite abundance in the principal host was found in the treatment with three native alternative hosts (1AB × 3RR; GLM, post hoc comparison, *p* = 0.011), but not in treatment with three non‐native alternative hosts (1AB × 3LG; GLM, post hoc comparison, *p* = 0.113; Figure 7A).

Nevertheless, the overall proportion of established parasites was lower in both treatments (χ = 209.0 and 276.7 for 1AB × 3RR and 1AB × 3LG, *p* < 0.001 for both tests) when compared with the conspecific (control) group (4AB), that is, the proportion of established parasites decreased from 82.5% (4AB) to 21.8% (1AB × 3RR) and 18.3% (1AB × 3LG). Parasite abundance of *R. rutilus* reached in the conspecific (control) group (4RR) did not change after adding three principal hosts (1RR × 3AB; GLM, post hoc comparison, *p* = 0.130) but decreased when adding the non‐native alternative hosts (1RR × 3LG; GLM, post hoc comparison, *p* = 0.010; Figure 7B). While the proportion of established parasites increased in 1RR × 3AB (33.6%; χ = 18.4, *p* < 0.001) compared with the conspecific group (4xRR; 19.3%), it decreased to 5% (χ = 43.1, *p* < 0.001) in the treatment with three *L. gibbosus* .

### 3.5 Host‐Related Parasite Size

**FIGURE 8:**
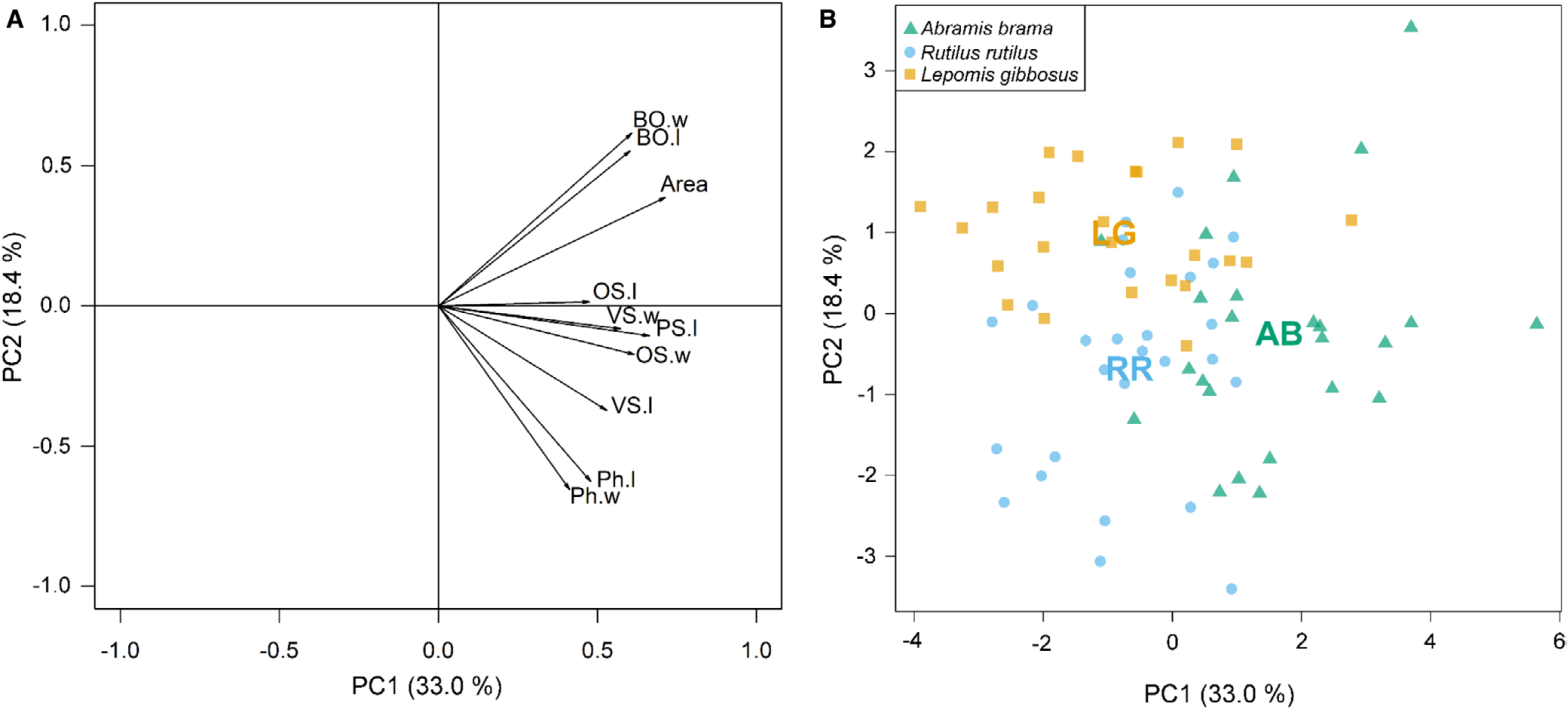
Association of morphological characteristics of *Diplostomum pseudospathaceum* with the first two PCA axes. Proportion of variability explained by each axis is shown in parenthesis. (A) Area—area of the metacercariae body, OS.l—oral sucker—length, OS.w—oral sucker—width, Ph.l—pharynx—length, Ph.w—pharynx—width, VS.l—ventral sucker—length, VS.w—ventral sucker—width, HO.l—holdfast organ—length, HO.w—holdfast organ—width, PS.l—pseudosuckers—mean length. (B) Positions of parasites from *Abramis brama* (AB, green), *Rutilus rutilus* (RR, blue) and *Lepomis gibbosus* (LG, yellow). Group centroids are marked by acronym.

The first PCA axis explained 33.0% of variability and represented an increasing gradient in all morphological variables, most strongly with length and width of oral and ventral sucker, length of pseudosuckers and body area (Figure 8A). The second axis explained 18.4% of variability and was associated mostly with length and width of the holdfast organ (positively) and pharynx length and width (negatively). Inter‐host differences in morphology of metacercariae were significant on both PCA axes (ANOVA, *F*_2,72_ = 23.44 and 13.56, both *p* < 0.001). Parasites recovered from *A. brama* were shifted significantly more to the right on the first PCA axis, compared with both *R. rutilus* and *L. gibbosus* (ANOVA, post hoc comparisons, both *p* < 0.001), which did not differ between each other (ANOVA, post hoc comparison, *p* = 0.826). The second PCA axis distinguished between parasites from *L. gibbosus* (shifted more to positive side of axis 2, toward higher values of holdfast organ and low values pharynx size) and two native species (ANOVA, post hoc comparisons, *p* = 0.001 and < 0.001 for *A. brama* and *R. rutilus* ; Figure 8), positions of which were not significantly different from each other (ANOVA, post hoc comparison, *p* = 0.392).

MANOVA supported PCA results, showing that parasite morphometry differed significantly between all three host species (MANOVA, approx. *F*_2,72_ = 7.64, *p* < 0.001; pairwise MANOVAs: *A. brama* vs. *R. rutilus* , approx. *F*_1,49_ = 7.60, *p* < 0.001, *A. brama* vs. *L. gibbosus* , approx. *F*_1,46_ = 11.26, *p* < 0.001, *L. gibbosus* vs. *R. rutilus* , approx. *F*_1,49_ = 10.88, *p* < 0.001; Table 2, Table 3). Generally, parasites recovered from the principal host *A. brama* tend to have longer and wider internal structures compared to two other species (with exception of length of oral sucker and pharynx, Figure 8; Table 2, Table 3), with both *A. brama* and *L. gibbosus* parasites having larger body area than those recovered from *R. rutilus* (ANOVA, *F*_2,72_ = 5.77, *p* = 0.005; post hoc comparisons, *p* = 0.033 and 0.006 for *R. rutilus* vs. *L. gibbosus* and *A. brama* , *p* = 0.825 for *L. gibbosus* vs. *A. brama* ).

**TABLE 2:** Measurements of area (in μm^2^) and dimensions (in μm) of internal organs of *Diplostomum pseudospathaceum* (mean ± SD) recovered from *Abramis brama* , *Rutilus rutilus* , and *Lepomis gibbosus* at 60 DPI within individual infection setup.

	*Abramis brama*	*Rutilus rutilus*	*Lepomis gibbosus*
	*N* = 24	*N* = 27	*N* = 24
Area	57,130 ± 5670	52,511 ± 4400	56,245 ± 5179
Oral sucker—length	41 ± 3	41 ± 2	40 ± 3
Oral sucker—width	44 ± 3	42 ± 2	40 ± 2
Pharynx—length	29 ± 3	28 ± 3	27 ± 3
Pharynx—width	27 ± 3	26 ± 3	24 ± 2
Ventral sucker—length	36 ± 3	35 ± 2	32 ± 2
Ventral sucker—width	44 ± 3	40 ± 3	40 ± 3
Holdfast organ—length	74 ± 7	68 ± 5	72 ± 5
Holdfast organ—width	76 ± 7	68 ± 7	74 ± 6
Pseudosuckers—length	40 ± 2	38 ± 2	37 ± 2

**TABLE 3:** Mean morphological parameter estimates in *Diplostomum pseudospathaceum* metacercariae recovered from three host fish species (AB – *Abramis brama* , RR – *Rutilus rutilus* , LG – *Lepomis gibbosus* ), standardized as ratio between the group estimate and the group with highest estimate of the parameter (AB for all parameters). *F*—*F*‐value of ANOVA; *p*—*p*‐value of ANOVA, and *p*‐values of multiple comparison Tukey HSD tests (AB × RR, AB × LG and RR × LG). Significant differences in bold.

	AB	RR	LG	*F*	*P*	AB × RR	AB × LG	RR × LG
Area	1.00	0.92	0.98	5.77	**0.005**	**0.006**	0.825	**0.033**
Oral sucker—length	1.00	0.99	0.97	0.91	0.408	0.779	0.374	0.757
Oral sucker—width	1.00	0.96	0.90	18.82	**0.000**	**0.022**	**0.000**	**0.002**
Pharynx—length	1.00	0.97	0.94	2.90	0.062	0.582	0.050	0.314
Pharynx—width	1.00	0.95	0.89	7.22	**0.001**	0.249	**0.001**	0.065
Ventral sucker—length	1.00	0.95	0.88	19.98	**0.000**	**0.036**	**0.000**	**0.001**
Ventral sucker—width	1.00	0.91	0.92	16.26	**0.000**	**0.000**	**0.000**	0.757
Holdfast organ—length	1.00	0.92	0.97	6.52	**0.003**	**0.002**	0.380	0.080
Holdfast organ—width	1.00	0.89	0.97	11.61	**0.000**	**0.000**	0.515	**0.003**
Pseudosuckers—length	1.00	0.95	0.92	15.45	**0.000**	**0.003**	**0.000**	0.063

## 4 Discussion

### 4.1 Host Susceptibility to a Generalist Parasite

Although *D. pseudospathaceum* is widely recognized as a generalist capable of infecting a broad spectrum of fish hosts across phylogenetically distant groups (e.g., Kudlai et al. 2017; Locke et al. 2020; Unger et al. 2022), our findings reveal a clear pattern of non‐random host selection, at least within restricted fish communities. Both field and experimental data consistently show that among fish species commonly inhabiting small lentic water bodies in Europe, certain hosts, particularly *A. brama* in our study, are highly infected, while others such as *R. rutilus* and the non‐native *L. gibbosus* exhibit significantly lower susceptibility. This pattern aligns with growing evidence that generalist parasites may still exhibit strong differences in host susceptibility, driven by variation in host quality rather than their availability (Manzoli et al. 2021), and that the terms specialist and generalist are context dependent and may not accurately describe the niche breadth of parasite taxa (Beasley 2024).

In our study, *A. brama* was not only the most heavily infected species in natural conditions but also showed consistently highest intensities of infection in controlled experimental setups, regardless of the density or presence of alternative hosts. Even when cercariae were exposed to equal numbers of different hosts, they disproportionately infected *A. brama* , suggesting active selection based on host competence. Our data thus align with the view that evolutionary compatibility and host–parasite coadaptation in generalists play a more significant role in shaping infection patterns than ecological factors such as host abundance or encounter rates (Locke et al. 2010; Rellstab et al. 2011). This is consistent with findings from other systems, where motile parasites such as trematode cercariae (Sears et al. 2012; Johnson et al. 2019) and ectoparasitic mites (Christe et al. 2007) have demonstrated adaptive host selection based on survival and transmission success.

Corresponding patterns can be found in newly established host–parasite interactions, offered by introductions of novel hosts. The susceptibility of these newly introduced hosts to local generalist parasites depends on the compatibility between parasite and host; some non‐native species are highly susceptible and suffer strong negative effects, while others are poor hosts, reducing parasite transmission or acting as dead‐end hosts (Prider et al. 2009; Hohenadler et al. 2019). The non‐native host *L. gibbosus* showed the lowest abundance of *D. pseudospathaceum* in both experimental and natural conditions. Interestingly, when a natural host was added in an experimental setup, infection levels in *L. gibbosus* increased, possibly due to chemical cues or behavioral stimulation triggered by the presence of a compatible host (Buchmann and Lindenstrøm 2002; Núñez‐Acuña et al. 2018). This suggests that host attractiveness for the parasite may be modulated by ecological context (Beasley 2024), and that parasite activation may be, to some extent, influenced by environmental or interspecific signals (Loot et al. 2006; Zhou et al. 2024). Moreover, host attractiveness and competence appear to be decoupled, with suboptimal hosts benefiting from proximity to more suitable ones (Johnson et al. 2019). Our findings indicate that variation in host susceptibility to a generalist parasite reflects a complex interplay of ecological opportunity and evolutionary adaptation that has important implications for understanding parasite transmission dynamics, especially in changing host communities related to the presence of invasive species.

### 4.2 Infection Success and the Dilution Effect

Host community composition, especially the presence and density of alternative hosts, can significantly alter parasite transmission dynamics (Searle et al. 2016; Buck et al. 2017). Presence of less competent host leads to the reduction of parasite transmission, usually through mechanisms such as the dilution effect and encounter reduction, with the density of alternative hosts being of particular importance (Civitello et al. 2015; Buck et al. 2017). The infection success in experimental setups significantly differed between the three host species tested, and the highest proportion of successfully established metacercariae was consistently found in the principal host, *A. brama* , irrespective of the host density or the proportion of additional alternative hosts. These results further confirm that *D. pseudospathaceum* encounters pronounced differences in host susceptibility and that host competence rather than density plays a central role in its infection success, consistent with previous studies on trematode systems (Johnson et al. 2019; Sears et al. 2012).

In multi‐host systems, the addition of a non‐native alternative host to the principal host (1:1) reduced the overall proportion of established parasites in the treatment by 17%, though no effect on infection success was observed after the addition of a native alternative host. Furthermore, this trend was more apparent in high‐density treatments (1:3). The addition of three alternative hosts (either native or non‐native) to a single principal host reduced the overall proportion of established parasites by 60%–64%. Notably, a dramatic drop in parasite infection success was apparent in *R. rutilus* when co‐exposed with three *L. gibbosus* , in which treatment only 5% of cercariae established successfully. These results align with the encounter‐dilution hypothesis predicting that increasing host density, particularly of low‐competence or non‐native hosts, can reduce per‐host infection risk by dispersing transmission stages among a larger number of potential hosts (Keesing et al. 2006; Buck and Lutterschmidt 2017).

The strong dilution effect exerted by non‐native *L. gibbosus* suggests that evolutionary novelty may enhance the diversion of parasite transmission away from native hosts. Cercariae may have failed to recognize the host's low suitability and waste transmission stages on it, as observed in other systems where invasive species disrupt the native host–parasite interactions (Kopp and Jokela 2007; Thieltges et al. 2009; Westby et al. 2019). Significant reduction in established parasites following the addition of *L. gibbosus* to the native hosts mirrors findings in amphibian systems, where non‐native hosts such as *Rana catesbeiana* Shaw, 1802 attracted a disproportionate number of cercariae but supported few successful infections, functioning as epidemiological sinks (Johnson et al. 2019). Similarly, treefrogs with strong immune defences reduced transmission to more competent hosts via encounter reduction (Johnson et al. 2008; Johnson and Hartson 2009). In our case, the immune response of *L. gibbosus* , evidenced by cataract formation, may contribute to its role as a low‐competence host that deflects parasites away from native species. Decline of abundance of *Diplostomum* spp. in natural fish hosts was observed in the St. Lawrence River following the introduction of non‐native Ponto‐Caspian gobies, suggesting that non‐native fish served as parasite decoys, diluting the infection (Gendron and Marcogliese 2017). Results of our experimental study further support this suggestion, showing that introduction of less competent host significantly reduces the infection in the parasites' natural hosts. Furthermore, disruption of the parasite life cycle due to limited transmission to the definitive bird host was found in the upper Paraná River basin, where the invasive pirarucu ( *Arapaima gigas* Cuvier, 1817) acts as an epidemiological sink for the eye metacercariae of *Austrodiplostomum compactum* (Lutz, 1928) Dubois, 1970 (Franceschini et al. 2025). The high density of the non‐native hosts capable of diluting parasitic infection, such as *L. gibbosus* with *D. pseudospathaceum*, will further influence the success of infection by such a parasite in a host community.

### 4.3 Cataract Intensity and Host Competence

Development of cataracts is the most prominent pathology of fish lens associated with the infection with metacercariae of *Diplostomum* spp. The severity of cataracts primarily reflects the number of parasites and the size of the fish (Karvonen et al. 2004), but there is a great variance among various host species as well as among conspecific individuals (Seppälä et al. 2011; Karvonen 2012). Our results revealed that *L. gibbosus* , the introduced host not co‐evolved with *D. pseudospathaceum*, exhibited a generally stronger reaction to infection expressed as cataract occurrence and severity compared to both the primary and the alternative native hosts (Figure 4). Notably, the probability of host response was higher in the introduced species even at lower infection intensities (Figure 5), indicating a heightened sensitivity to the infection. The severe pathology was observed even in fish infected with a low number of parasites (1–2), resulting in cataract score 2–3. Although this response is common in small fish (Karvonen and Lindström 2018), which were used in this study, only introduced *L. gibbosus* responded with such a severe cataract formation. On the other hand, metacercariae in both native species, *A. brama* and *R. rutilus* , induced cataracts at higher infection intensities (11 and 4–5, respectively) with generally lower cataract scores (mostly 1). Cataract formation in our experiment did not correlate with host competence (primary vs. alternative host) but rather appeared to reflect a novel parasite–host interaction in the introduced species, which may lack adaptive mechanisms to mitigate the effects of infection.

Recently established parasites do not usually cause strong cataracts. Instead, cataracts mainly develop when the metacercariae are reaching full development (Seppälä et al. 2005). Cataract severity was measured at two time points, covering the initial phase of metacercarial maturity (30 dpi) and fully developed parasites (60 dpi) (Seppälä et al. 2004). During the metacercarial maturation phase, the frequency of host reaction was significantly higher in the introduced species than in native hosts. However, this difference diminished over time (a month later), when 75%–81% of fish exhibited cataract irrespective of the host species. Nevertheless, the severity of reaction remained consistently higher in the introduced host throughout the chronic phase. Novel hosts for the parasite often mount a more pronounced response to the infection than adapted hosts, and the lack of co‐evolved defences may lead to higher susceptibility and/or more severe pathology. For example, European eels (novel hosts) infected with the nematode *Anguillicola crassus* Kuwahara, Niimi & Itagaki, 1974, showed a larger and more functionally diverse set of differentially expressed genes than Japanese eels (adapted hosts). The adapted host's immune response was more limited and transient, while the novel host's response was sustained and involved more physiological disruption (Bracamonte et al. 2019).

In spite of the fact that metacercariae of *D. pseudospathaceum* are situated in an immune‐privileged organ (eye), recent studies showed that the parasite may provoke the fish's immune response (Haase et al. 2014; Fuad et al. 2024) and the immunocompetence reflects the host population‐level adaptation (Kalbe and Kurtz 2006). Adapted hosts may rely on targeted, efficient immune responses or tolerance mechanisms, while novel hosts may experience widespread physiological disruption, including energetically costly altered metabolism and stress responses (Mandl et al. 2015; Bracamonte et al. 2019). Although the immune response was not measured in this study, all three host species exhibited a trend of decreasing cataract severity over time, suggesting the activation of repair mechanisms or physiological adaptation to infection (Karvonen et al. 2004; Ondračková, Valigurová, et al. 2025b). This pattern may reflect host plasticity or immune modulation aimed at minimizing long‐term damage from parasitic presence.

### 4.4 Parasite Morphometrical Variability

Metacercariae of *D. pseudospathaceum* exhibited subtle but significant variation in size and dimensions of internal organs depending on the host species. The experimental design minimized confounding factors such as parasite genetic affiliation (Kalbe and Kurtz 2006), environmental conditions such as water temperature, age of the parasite (Mironova et al. 2022), and size/age of the host (Poulin et al. 2003). Phenotypic variation in digeneans can be influenced by the intensity of infection (“crowding effect”, Fredensborg and Poulin 2005) and the host identity (“host‐induced variation”; Niewiadomska and Szymanski 1992), and the intra‐host competition generally induces a negative relationship between individual parasite size and parasite load (Fredensborg and Poulin 2005; Sistermans et al. 2023). On the other hand, density‐dependent increase in size of the parasite (Alee effect) was observed in *D. pseudospathaceum* from experimentally infected *Oncorhynchus mykiss* (Walbaum, 1792) (Mironova et al. 2022). In accordance with their study, most measured traits reached significantly larger dimensions in the principal host which exhibited the intensity of infection more than twice as high compared to less infected alternative hosts. However, when testing the association between intensity of infection and parasite size separately per host species (corresponding to Mironova et al. 2022), such results were not found (see Appendix S2 for additional details).

In helminths, larger larval sizes in intermediate hosts are often associated with fitness benefits in definitive hosts, including increased infectivity, establishment success and better survival (Rosen and Dick 1983; Steinauer and Nickol 2003; Benesh and Hafer 2012), as well as larger adult body size and higher fecundity (Fredensborg and Poulin 2005). Achieving larger size, coupled with significantly higher transmission success, as observed for parasites developing in the principal host in this study, may therefore make them more likely to complete their life cycle successfully. Thus, even subtle host‐related differences in larval morphology, which were observed in our study, may be relevant for later stages of the parasite life cycle, given the above‐mentioned links between larval size and fitness in definitive hosts, and supporting the idea that intermediate host selection is a critical component of parasite life cycle optimization (Parker et al. 2009).

### 4.5 Limitations of This Study

Despite providing clear support for dilution of a generalist parasite following the non‐native host introduction, our study has several limitations. This study was conducted under controlled experimental conditions, which may not fully replicate the complexity of natural environments. By standardizing fish size and age (0+), we controlled for confounding factors such as surface area and immune development, which are known to affect parasite abundance (Poulin 1999, 2000). However, in the wild, additional ecological and physiological factors, such as host immune responses, environmental stressors, and other parasite–host interactions, could influence the infection success. Furthermore, *Diplostomum* spp. are known to exhibit genotypic variation that can influence host specificity and infection success in different host species (Kalbe and Kurtz 2006; Locke et al. 2010). To minimize genotype‐specific effects, we used a mix of cercariae from multiple snails, also to reflect the natural diversity encountered by fish in the wild. We also acknowledge that our experimental design, which combined a maximum of two host species at a time, does not fully reflect the complexity of natural host communities (Ahn and Goater 2021). Nevertheless, this simplified approach allowed us to isolate and demonstrate the specific impact of invasive hosts on parasite establishment success, thereby providing valuable insights into host identity as a key factor shaping parasite dynamics.

## 5 Conclusions

Our experimental study demonstrated that the introduction of a non‐native less‐competent host may significantly reduce the infection success of a generalist parasite. In to previous experimental studies using non‐competent or resistant host species (Kopp and Jokella 2007, Thieltegs et al. 2009), our results show that dilution effects are not limited to non‐competent hosts. Even susceptible but less competent native hosts can reduce parasite success when present in sufficient numbers, supporting the notion that host competence exists on a continuum (Holmes and Price 1986; Gervasi et al. 2015; Martin et al. 2019). This finding has significant implications for understanding parasite transmission in changing communities, where shifts in host abundance or the introduction of novel species can alter infection dynamics in unpredictable ways. In conclusion, our study demonstrates that host density, competence, and evolutionary history jointly shape infection success in multi‐host parasite systems. The presence of alternative hosts, especially the non‐native ones, can reduce parasite transmission through encounter dilution and suboptimal infection, even when those hosts are susceptible. These findings underscore the importance of integrating host susceptibility, competence, and community structure when predicting disease risk and managing parasite transmission in natural ecosystems.

## Author Contributions

**Markéta Ondračková:** conceptualization (lead), data curation (lead), funding acquisition (lead), investigation (equal), methodology (equal), visualization (equal), writing – original draft (lead), writing – review and editing (equal). **Lukáš Vetešník:** investigation (supporting), funding acquisition (equal), writing – review and editing (supporting). **Veronika Bartáková:** formal analysis (equal), writing – review and editing (supporting). **Michal Janáč:** formal analysis (lead), visualization (equal), writing – review and editing (equal).

## Funding

This study was supported through the Czech Science Foundation, project No. 23‐07185S.

## Ethics Statement

This research was undertaken in line with the ethical requirements of the Czech Republic and has been approved by the appropriate ethics committee (permits No. UBO‐522/Sekr‐c/2023 MZP/2023/630/2770 and UBO‐718/Sekr‐c/2023 AVCR 9083/2022). The method of fish sampling, experimental infection, and killing complied with the legal requirements of the Czech Republic (§ 7 law No. 114/1992 on the Protection of Nature and Landscape and § 6, 7, 9 and 10 regulation No. 419/2012 on the Care, Breeding and Use of Experimental Animals).

## Conflicts of Interest

The authors declare no conflicts of interest.

## Supporting information


**Appendix S1:** List of fish species examined for the presence of *Diplostomum* metacercariae in lentic water bodies of the lower Morava and Dyje Rivers, Czech Republic, with number fish collected, prevalence of *Diplostomum* infection, and species found in particular tissue locations. Note that only a subsample of parasites found in each host species was used for *Diplostomum* identification by molecular methods.
**Appendix S2:** Results of Spearman rank correlation analyses testing associations between the intensity of infection and size of internal organs of *Diplostomum pseudospathaceum*. Bonferroni correction applied to adjust significance levels and reduce the probability of committing a Type I error established the significance level at *p* < 0.005. Significant differences in bold.

## Data Availability

The data that support the findings of this study are privately available via Figshare—https://figshare.com/s/8f1d47962222dc71049f.
