# Supplementary material for: Competent Non‐Native Alternative Host Reduces Infection Success in a Generalist Parasite
Source: Ecol Evol. 2026 May 27;16(6):e73645. doi: 10.1002/ece3.73645 (PMC13239089; doi:10.1002/ece3.73645)
Supplement: Supplementary file 1 — Appendix S1: List of fish species examined for the presence of Diplostomum metacercariae in lentic water bodies of the lower Morava and Dyje Rivers, Czech Republic, with number fish collected, prevalence of Diplostomum infection, and species found in particular tissue locations. Note that only a subsample of parasites found in each host species was used for Diplostomum identification by molecular methods. Appendix S2: Results of Spearman rank correlation analyses testing associations between the intensity of infection and size of internal organs of Diplostomum pseudospathaceum. Bonferroni correction applied to adjust significance levels and reduce the probability of committing a Type I error established the significance level at p < 0.005. Significant differences in bold. [file ECE3-16-e73645-s001.docx]

**Supporting Information for:**

**Competent non-native alternative host reduces infection success in a generalist parasite**

**Appendix S1**

| **Appendix S1.1** | Page 1 |
| --- | --- |
| **Appendix S1.2** | Page 2 |
| **Appendix S1.3** | Page 3 |

**Appendix S1.1**. List of fish species examined for the presence of *Diplostomum* metacercariae in lentic water bodies of the lower Morava and Dyje Rivers, Czech Republic, with number fish collected, prevalence of *Diplostomum* infection, and species found in particular tissue locations. Note that only a subsample of parasites found in each host species was used for *Diplostomum* identification by molecular methods.

| **Fish host species** | **N fish** | **Prevalence** | **Tissue location** | ***Diplostomum* species** |
| --- | --- | --- | --- | --- |
| *Abramis brama* | 12 | 33% | lens | *D. pseudospathaceum, D. spathaceum* |
| *Rutilus rutilus* | 140 | 18% | lens | *D. mergi, D. pseudospathaceum, D. spathaceum* |
| *Scardinius erythrophthalmus* | 142 | 4% | lens | *D. pseudospathaceum, D. spathaceum* |
| *Perca fluviatilis* | 136 | 24% | vitreous humour | *Diplostomum* sp. lineage 3/4* |
| *Gymnocephalus cernuus* | 42 | 24% | vitreous humour | *Diplostomum* sp. lineage 3/4* |
|  |  |  | lens | *D. pseudospathaceum, D. spathaceum* |
| *Lepomis gibbosus* | 141 | 10% | lens | *D. pseudospathaceum* |
| *Pseudorasbora parva* | 76 | 1% | lens | *D. spathaceum* |
| *Carassius gibelio* | 42 |  | no parasites found |  |

* *Diplostomum* from vitreous humour *P. fluviatilis* and *G. cernuus* showed affiliation to *Diplostomum* sp. lineage 3 sensu Faltýnková et al. (2022) according to ITS1-5.8S-ITS2 rRNA and *Diplostomum* sp. lineage 4 sensu Faltýnková et al. (2022) according to COI.

**Appendix S1.2.** Results of Spearman rank correlation analyses testing associations between the intensity of infection and size of internal organs of *Diplostomum pseudospathaceum*. Bonferroni correction applied to adjust significance levels and reduce the probability of committing a Type I error established the significance level at *P* < 0.005. Significant differences in bold.

|  | *A. brama* | | *R. rutilus* | | *L. gibbosus* | |
| --- | --- | --- | --- | --- | --- | --- |
|  | N = 24 | | N = 27 | | N = 24 | |
|  | *r_s_* | *P* | *r_s_* | *P* | *r_s_* | *P* |
| Area | -0.533 | 0.007 | 0.092 | 0.646 | -0.304 | 0.148 |
| Oral sucker - length | -0.279 | 0.187 | 0.316 | 0.108 | -0.190 | 0.374 |
| Oral sucker - width | -0.316 | 0.133 | 0.112 | 0.578 | -0.052 | 0.808 |
| Pharynx - length | 0.047 | 0.828 | -0.445 | 0.020 | -0.133 | 0.535 |
| Pharynx - width | -0.200 | 0.348 | -0.405 | 0.036 | -0.058 | 0.787 |
| Ventral sucker - length | 0.278 | 0.189 | -0.084 | 0.678 | -0.074 | 0.733 |
| Ventral sucker - width | -0.171 | 0.426 | 0.316 | 0.108 | 0.003 | 0.988 |
| Holdfast organ - length | -0.207 | 0.331 | 0.212 | 0.288 | -0.042 | 0.844 |
| Holdfast organ - width | -0.298 | 0.158 | 0.229 | 0.250 | -0.393 | 0.057 |
| Pseudosuckers - length | 0.505 | 0.012 | 0.196 | 0.326 | **-0.572** | **0.004** |
